# Prenatal Diagnosis and Postnatal Follow‐Up of 19 Fetuses With 16p13.11 Microduplication in a Cohort of 12,038 Pregnancies From South Zhejiang

**DOI:** 10.1002/mgg3.70324

**Published:** 2026-10-15

**Authors:** Xiaoxiao Lv, Shufei Li, Lili Zhou, Ruipu Wang, Yuan Yu, Chenyang Xu, Xueqin Xu, Xiaohua Tang, Huanzheng Li

**Affiliations:** ^1^ Wenzhou Central Hospital Wenzhou China

**Keywords:** 16p13.11 microduplication, CNVs, phenotype, prenatal diagnosis

## Abstract

**Background:**

Prenatal interpretation of 16p13.11 microduplication remains difficult in daily practice, mainly because the same duplication may be associated with very different clinical outcomes. In this study, we reviewed fetuses diagnosed with 16p13.11 microduplication at our center and summarized their prenatal findings, pregnancy outcomes, and postnatal development to provide data that may be useful in prenatal counseling.

**Methods:**

We retrospectively reviewed 12,038 pregnant women who underwent amniocentesis at Wenzhou Central Hospital between 2014 and 2025. Chromosomal microarray analysis was performed in all cases. For fetuses carrying 16p13.11 microduplication, we reviewed the indication for amniocentesis, ultrasound findings, pregnancy outcome, and available postnatal follow‐up information.

**Results:**

Chromosomal microarray analysis (CMA) identified 16p13.11 microduplication in 19 fetuses, with duplication sizes ranging from 0.80 to 1.60 Mb. The main indications for prenatal diagnosis were advanced maternal age (*n* = 7), adverse pregnancy history (*n* = 5), ultrasound abnormalities (*n* = 4), and high‐risk serum screening results (*n* = 3). Three pregnancies were terminated. Sixteen infants were live‐born, of whom two were lost to follow‐up. Among the remaining 14 children, the follow‐up period ranged from 10 months to 10 years. One infant was hospitalized at birth for a ventricular septal defect, and one child showed growth and developmental delay. Five children showed better growth and development than their peers, whereas seven had no apparent abnormalities during follow‐up.

**Conclusion:**

The follow‐up results in our cohort are consistent with the incomplete penetrance and variable outcomes reported for 16p13.11 microduplication. In this cohort, most live‐born children showed mild or no clinical manifestations. In prenatal counseling, this CNV should be discussed together with fetal imaging results, inheritance pattern, family history, and available follow‐up data rather than interpreted as an isolated finding.

## Graphical abstract

In this prenatal cohort, 16p13.11 microduplication was associated with variable outcomes and incomplete penetrance. Most live‐born children showed mild or no abnormalities, highlighting the need for cautious interpretation and individualized genetic counseling.

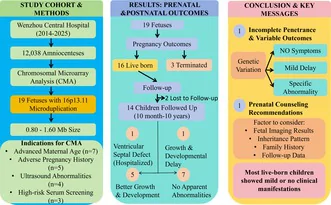

## 1 Introduction

The 16p13.11 region is a genomically unstable segment enriched with low‐copy repeats (LCRs). This genomic architecture predisposes the region to replication errors and non‐allelic homologous recombination, making it a recurrent hotspot for copy number variations (CNVs) and contributing to the pathogenesis of several genomic disorders (Loftus et al. 1999; Ullmann et al. 2007). Reported clinical manifestations associated with 16p13.11 CNVs include developmental delay (DD), intellectual disability (ID), language impairment, behavioral abnormalities, dysmorphic facial features, attention‐deficit/hyperactivity disorder (ADHD), autism spectrum disorder (ASD), and cardiovascular anomalies (Hamad et al. 2023). Among these variants, 16p13.11 microduplication is a rare CNV characterized by marked phenotypic variability and incomplete penetrance. Because this duplication is often inherited from apparently unaffected parents, its clinical significance remains difficult to interpret and continues to be debated.

Most published studies on 16p13.11 microduplication have focused on pediatric or adult populations (Brownstein et al. 2016), whereas data on prenatal detection and postnatal follow‐up remain limited. The association between prenatal ultrasound findings and postnatal outcomes in fetuses carrying 16p13.11 microduplication is still not well defined. In addition, long‐term follow‐up data from Chinese populations, particularly single‐center prenatal cohorts, are scarce. As 16p13.11 microduplication is increasingly detected during prenatal CNV screening, its limited fetal phenotypic manifestations and incomplete penetrance pose substantial challenges for variant interpretation and prenatal genetic counseling.

In this study, we retrospectively analyzed 19 fetuses diagnosed with 16p13.11 microduplication by chromosomal microarray analysis (CMA). By integrating prenatal ultrasound findings, pregnancy outcomes, and postnatal follow‐up data, we aimed to evaluate the genotype–phenotype correlation of this CNV in the prenatal setting. We hope that these follow‐up data will be useful when counseling families in whom this duplication is detected prenatally.

## 2 Materials and Methods

### 2.1 Patient Recruitment and Ethical Statement

We retrospectively reviewed 12,038 fetuses who underwent prenatal testing using amniotic fluid samples at Wenzhou Central Hospital from 2014 to 2025. Maternal age ranged from 19 to 54 years, and gestational age at amniocentesis ranged from 16 to 24 weeks. The main indications for testing were advanced maternal age, high‐risk serum screening results, ultrasound abnormalities, adverse pregnancy history, and other clinical indications. All pregnant women provided written informed consent before testing. This study was approved by the Institutional Research Ethics Committee of Wenzhou Central Hospital (No. L2026‐05‐031).

### 2.2 DNA Extraction

Fetal genomic DNA was extracted from uncultured amniotic fluid cells using a genomic DNA extraction kit (Kaishuo Biotechnology, China) according to the manufacturer's instructions. For cases requiring pedigree analysis, peripheral blood samples were collected from both parents, and genomic DNA was isolated using the same protocol. DNA concentration and purity were assessed using a NanoDrop 2000 UV–Vis spectrophotometer (Thermo Fisher Scientific, USA). Only DNA samples with a concentration of 3–25 ng/μL and an A260/A280 ratio of 1.7–2.0 were used for subsequent analysis. Qualified DNA samples were stored at −20°C until further use.

### 2.3 Chromosomal Microarray Analysis

The American College of Obstetricians and Gynecologists (ACOG) and the Society for Maternal‐Fetal Medicine (SMFM) published joint recommendations regarding the use of microarray analysis in prenatal diagnosis(“(Committee Opinion No. 581 2013): The use of chromosomal microarray analysis in prenatal diagnosis,”). The societies recommended CMA in lieu of karyotyping for patients who wished to proceed with prenatal diagnosis by chorionic villus sampling (CVS) or amniocentesis in the setting of one or more structural fetal anomalies identified by ultrasound. For patients who wished to pursue prenatal diagnosis in the setting of a normal fetal ultrasound, the societies recommend offering either CMA or karyotyping. Chromosomal microarray analysis was performed using the CytoScan 750 K Array platform (Affymetrix, Santa Clara, CA, USA) according to the manufacturer's protocol. Briefly, genomic DNA was subjected to digestion, ligation, PCR amplification, purification, quantification, fragmentation, labeling, hybridization to the array, washing, staining, and scanning. Raw data were processed for signal extraction and quality control, and copy number variants were analyzed using Chromosome Analysis Suite (ChAS) software.

### 2.4 Pathogenicity Analysis

CNVs were interpreted through a comprehensive evaluation based on multiple factors, including the number of protein‐coding genes involved, gene dosage sensitivity, records in public databases, parental origin, inheritance pattern of the associated disorder, previously published reports, and in‐house laboratory data (Firth et al. 2009; Rehm et al. 2015; Riggs et al. 2020). The main reference databases included the Database of Genomic Variants (DGV; http://dgv.tcag.ca/dgv/app/home), DECIPHER (http://decipher.sanger.ac.uk/), Online Mendelian Inheritance in Man (OMIM; http://www.omim.org), the ClinGen Dosage Sensitivity Map (https://www.ncbi.nlm.nih.gov/projects/dbvar/clingen), the University of California Santa Cruz Genome Browser (UCSC; https://genome.ucsc.edu/index.html), and ClinVar (https://www.ncbi.nlm.nih.gov/clinvar). According to the American College of Medical Genetics and Genomics (ACMG) standards and guidelines, CNVs were classified as pathogenic, likely pathogenic, variants of uncertain significance (VUS), likely benign, or benign. All reported CNV coordinates were based on the National Center for Biotechnology Information human genome build 37 (hg19).

### 2.5 Follow‐Up

For each pregnancy with a fetal 16p13.11 microduplication, we obtained pregnancy outcome and neonatal health information through telephone follow‐up and review of the electronic maternal health records. A structured follow‐up protocol was established, covering the neonatal period, infancy, and early childhood. Collected indicators included growth parameters, motor and cognitive development, language development, and social–behavioral abilities. Evaluations of the nervous, cardiovascular, and other organ systems were also included during follow‐up.

Developmental assessments were performed with reference to the Wechsler Intelligence Scale for Children, the Neuropsychological Development Scale for Children Aged 0–6 Years, and the Gesell Developmental Schedule, as appropriate for age. Height and weight were evaluated according to the child growth curves provided in the 8th edition of Pediatrics. The overall study workflow is shown in Figure 1.

**FIGURE 1:**
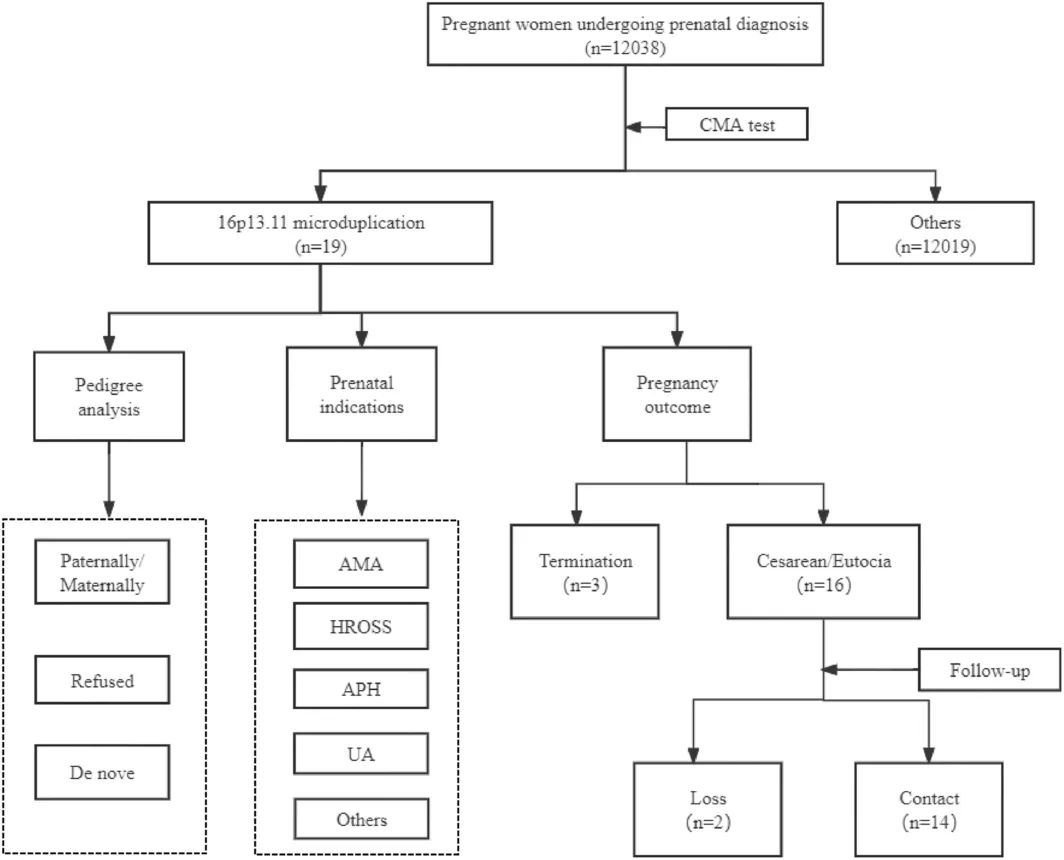
Research design flowchart. AMA, advanced maternal age; HROSS, high risk of serum screening; APH, adverse pregnancy history; UA, ultrasound abnormalities.

## 3 Results

### 3.1 Detection Rate and Genomic Characteristics of 16p13.11 Microduplication

In this study, 16p13.11 microduplication was identified in 19 fetuses, corresponding to a detection rate of 0.16% (19/12,038). The duplicated region extended from 14,920,864 to 16,520,463 bp according to the hg19/GRCh37 reference genome. Duplication sizes ranged from 0.80 to 1.60 Mb and encompassed 7–13 OMIM genes. All duplicated segments overlapped the critical region associated with 16p13.11 microduplication syndrome (Figure 2).

**FIGURE 2:**
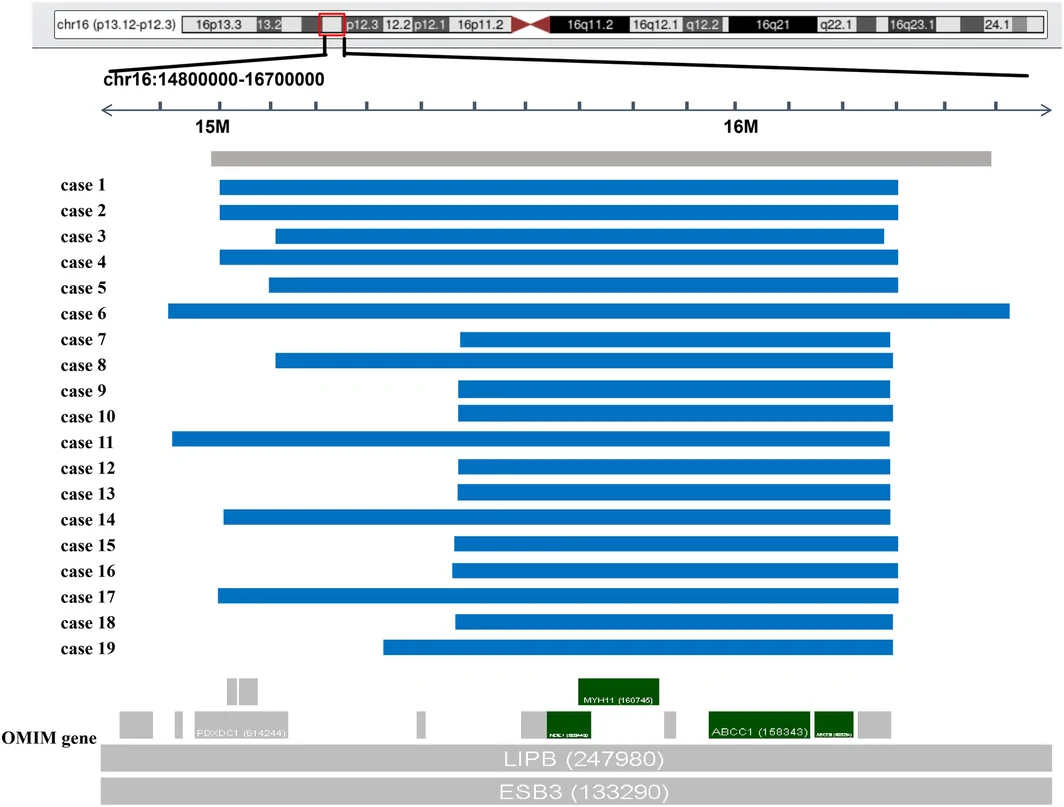
16p13.11 microduplication detected using CMA. The dark gray‐colored area indicates the 16p13.11 microduplication syndrome region. The blue bars indicate the location of duplication in the 16p13.11 region for 19 fetuses. OMIM genes are also color‐coded: Dark green for morbid genes with established clinical relevance, gray for all other OMIM genes.

### 3.2 Indications for Prenatal Diagnosis and Abnormal Prenatal Ultrasonographic Findings

Among the 19 fetuses with 16p13.11 microduplication, the indications for prenatal diagnosis were advanced maternal age in seven cases, adverse pregnancy history in five, abnormal ultrasound findings in four, and high‐risk serum screening results in three. The four cases with ultrasound abnormalities were as follows. Case 9 showed increased nuchal translucency (NT). Case 10 presented with multiple fetal anomalies, including fetal growth lagging approximately 3 weeks behind gestational age, bilateral pleural effusion, peritoneal effusion, cardiomegaly, and tricuspid regurgitation (Figure 3a). Case 11 showed right upper limb abnormalities suggestive of radial dysplasia, including a short and curved ulna, an extremely short radius, and marked angular deviation of the wrist (Figure 3b,c). Case 14 showed a single umbilical artery and an echogenic intracardiac focus (EIF) in the left ventricle (Figure 3d); this infant was subsequently hospitalized after birth because of a ventricular septal defect (VSD).

**FIGURE 3:**
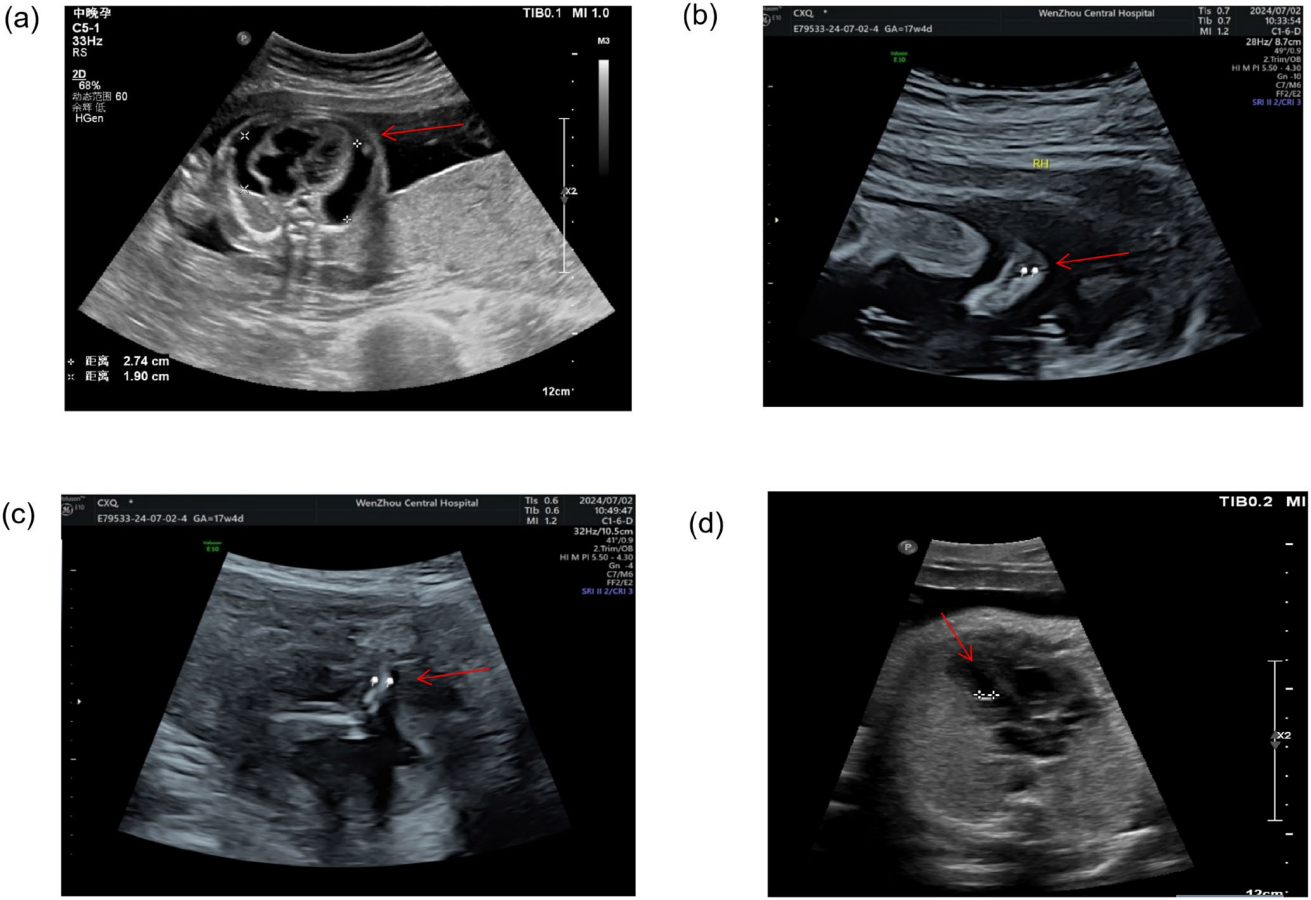
Ultrasound findings in Cases 10, 11, and 14. (a) Pleural effusion in Case 10. (b, c) Right upper limb deformity in Case 11. (d) Echogenic intracardiac focus in the left ventricle in Case 14.

### 3.3 Pedigree Analysis

Pedigree verification was performed in only five of the 19 fetuses (Table 1). Among these five cases, one microduplication occurred de novo, whereas the remaining four were inherited from either the mother (*n* = 2) or the father (*n* = 2).

**TABLE 1:** Prenatal indications, CMA results, and follow‐up information of 19 cases with 16p13.11 duplication.

Case	Pregnant woman age(Y)	Indication	CMA	Size(Mb)	OMIM gene	Inheritance	Pregnancy outcome	Gender	First words (months)	Walking independently (months)	Current age(Y/M)	Follow‐up information
1	35	AMA	15,052,746–16,308,351	1.25	10	Refused	Live birth	Boy	8	10	10Y	Superior to age peers
2	28	APH	15,052,746–16,308,351	1.25	10	Maternal	Live birth	Girl	12	12	8Y	No visible abnormalities
3	35	AMA	15,140,211–16,289,532	1.14	10	Refused	Live birth	Boy	12	12	10Y	Superior to age peers
4	34	APH	15,052,746–16,308,351	1.25	10	Refused	Live birth	Girl	36	17	9Y	Language and motor development delay
5	34	AMA	15,127,986–16,308,351	1.18	10	Refused	TP	—	—	—	—	N/A
6	40	AMA	14,920,864–16,520,463	1.60	13	Refused	Live birth	Girl	12	12	8Y	Superior to age peers
7	34	HROSS	15,481,747–16,278,133	0.80	7	Refused	Live birth	Boy	—	—	8Y	Loss to follow‐up
8	31	HROSS	15,154,356–16,282,869	1.13	9	Refused	Live birth	Girl	12	12	6Y	Superior to age peers
9	20	UA	15,481,747–16,278,133	0.80	7	*De novo*	Live birth	Boy	8	8	7Y	No visible abnormalities
10	28	UA	15,481,747–16,282,869	0.80	7	Refused	TP	—	—	—	—	N/A
11	30	UA	14,929,070–16,278,132	1.35	12	Refused	TP	—	—	—	—	N/A
12	40	AMA	15,481,747–16,282,869	0.80	7	Refused	Live birth	Girl	12	13	4Y	Superior to age peers
13	41	AMA	15,481,747–16,278,132	0.80	7	Paternal	Live birth	Boy	12	11	3Y	No visible abnormalities
14	40	UA	15,058,822–16,278,132	1.22	10	Refused	Live birth	Girl	15	15	3Y	VSD
15	34	HROSS	15,481,748–16,309,046	0.83	7	Maternal	Live birth	Girl	12	12	2Y	No visible abnormalities
16	24	APH	15,481,748–16,309,046	0.83	7	Refused	Live birth	Boy	12	12	2Y	No visible abnormalities
17	38	APH	15,052,746–16,308,351	1.25	10	Refused	Live birth	Boy	—	—	8Y	Loss to follow‐up
18	39	AMA	15,481,748–16,282,869	0.80	7	Refused	Live birth	Girl	—	—	10 M	No visible abnormalities
19	29	APH	15,338,153–16,278,132	0.94	7	Paternal	Live birth	Boy	—	—	10 M	No visible abnormalities

Abbreviations: AMA, advanced maternal age; APH, adverse pregnancy history; HROSS, high risk of serum screening; N/A, not applicable; TP, termination of pregnancy; UA, ultrasound abnormalities; VSD, ventricular septal defect.

### 3.4 Pregnancy Outcome and Follow‐Up Information

Three pregnancies (Cases 5, 10, and 11) were terminated after detailed genetic counseling. The remaining 16 fetuses were carried to term, and two were lost to follow‐up. Postnatal follow‐up was available for 14 children, with ages ranging from 10 months to 10 years. One infant was hospitalized at birth because of a VSD, but subsequent re‐examinations revealed no additional abnormalities. One 9 year‐old child showed delayed growth and development; this child began walking at 17 months and speaking at 3 years, and the parents reported average expressive language ability. Five children showed more advanced growth and development than age‐matched peers, whereas the parents of the remaining seven children reported no abnormalities (Table 1).

## 4 Discussion

In this study, 16p13.11 microduplication was identified in 19 of 12,038 pregnancies undergoing chromosomal microarray analysis, giving a detection rate of approximately 0.16%. The duplicated segments overlapped the recurrent 16p13.11 microduplication region and, according to Table 1, ranged from 0.80 to 1.60 Mb with 7–13 OMIM genes involved. After receiving multidisciplinary genetic counseling, three couples chose termination of pregnancy, whereas the remaining 16 pregnancies continued to live birth. Postnatal follow‐up was available for 14 children, and two were lost to follow‐up. Most followed children were reported by their parents to have no obvious abnormalities. One child had delayed language and motor development, and another was hospitalized after birth for ventricular septal defect. In 2024, ClinGen assigned a triplosensitivity score of 2 to this genomic region, suggestive of moderate pathogenic potential. These outcomes reflect the main difficulty encountered in prenatal counseling: the same recurrent duplication may be followed by either apparently normal development or clinically relevant abnormalities (Pan et al. 2025).

The 16p13.11 region is prone to recurrent rearrangement because of its low‐copy repeat structure (Slavotinek 2008). Duplications involving this region have been associated with a broad range of neurodevelopmental, behavioral, dysmorphic, and cardiovascular phenotypes (Hannes et al. 2009; Nagamani et al. 2011). At the same time, overlapping duplications have also been found in apparently unaffected individuals and in phenotypically normal parents (Zhao et al. 2024). Our cohort shows this same contrast: most followed children had no obvious abnormalities, whereas one child showed delayed walking and speech. This wide range of outcomes, from apparently normal development to developmental delay, supports the interpretation of 16p13.11 microduplication as a susceptibility CNV rather than a fully penetrant pathogenic variant.

Recent prenatal series have reported broadly similar outcomes. Xu et al., Cai et al., Zhao et al., and Luo et al. all found that most live‐born children with 16p13.11 duplication had no major abnormalities during early follow‐up, though neurodevelopmental abnormalities appeared in a minority (Cai et al. 2022; Luo et al. 2025; Xu et al. 2026; Zhao et al. 2024). Our data are consistent with this pattern. Most followed children had no apparent developmental or health problems according to parental report, while one child had language and motor developmental delay and another had ventricular septal defect after birth. These observations are reassuring for early childhood outcome, but they do not remove the need for longer follow‐up, since cognitive, behavioral, or neurodevelopmental problems may become more evident with age (Delicado et al. 2014). Pan et al. reported 14 fetuses with 16p13.11 microduplication identified among 4552 pregnancies undergoing amniocentesis and SNP‐array testing, with postnatal follow‐up ranging from 1 year and 1 month to 3 years (Pan et al. 2025). Their findings, as well as ours, illustrate the marked variability associated with this CNV: most children who were followed had no reported major abnormalities, whereas developmental delay and ventricular septal defect were observed in individual cases. The present series included 19 fetuses from a clinical cohort of 12,038 pregnancies and allowed follow‐up for up to 10 years. In addition to the variable postnatal outcomes, Cases 10 and 11 showed severe prenatal abnormalities affecting more than one organ system. These observations add to the limited prenatal literature on 16p13.11 microduplication and may be useful when counseling families. However, the number of cases remains small, parental testing was incomplete, and developmental outcomes were based mainly on parental reports; thus, the prognosis for an individual fetus cannot be predicted from the CNV result alone.

Prenatal ultrasound abnormalities were found in four fetuses, and the findings were heterogeneous. Case 9 had increased nuchal translucency. Case 10 showed fetal growth restriction, bilateral pleural effusion, ascites, cardiomegaly, and tricuspid regurgitation. Case 11 had right upper‐limb anomalies, including radial dysplasia, a shortened and curved ulna, an extremely short radius, and wrist angulation. Case 14 had a single umbilical artery and left ventricular echogenic focus; after birth, this child was hospitalized for ventricular septal defect. Structural abnormalities involving the heart and other organ systems have also been described in previous prenatal cohorts (Calderoni et al. 2020; Hamad et al. 2023). The findings in our cohort add to these observations, but they do not prove that the duplication caused the ultrasound abnormalities. In the terminated pregnancies, systematic fetal autopsy and additional genetic testing were not performed; therefore, other genetic or non‐genetic causes were not ruled out. These prenatal findings are best regarded as potentially associated manifestations.

The duplicated segments encompassed multiple OMIM genes. *NDE1* is of particular interest because of its role in neurogenesis and cortical development (Houlihan and Feng 2014). Case 4 carried a 1.25 Mb duplication involving 10 OMIM genes and had delayed walking and language development, which is compatible with the neurodevelopmental spectrum reported in 16p13.11 imbalance. The relationship, however, is not direct. Other children with overlapping duplications, including some with similar duplication sizes or gene counts, had no reported developmental abnormalities during follow‐up. This contrast within the same cohort shows that CNV size or gene content alone cannot reliably predict outcome. Background genetic factors and other modifiers probably contribute to the variability, but their individual effects cannot be determined from our data alone (Consortium 2012).

The contribution of other genes in the interval is less clear. Pathogenic variants in *ABCC6* cause abnormal mineralization and arterial calcification (Wang et al. 2026), but it is unclear what clinical effect, if any, extra copies of *ABCC6* produce. *MYH11* also sits within the recurrent region. One child (case 14) in our cohort had ventricular septal defect after birth, but our data do not support a direct link between *MYH11* duplication and cardiovascular abnormalities. Taken together, these genes offer biological context for clinical discussion, but they do not yet support reliable genotype–phenotype predictions.

The clinical value of this study lies mainly in prenatal counseling. When 16p13.11 microduplication is detected prenatally, it should not be presented to parents as either a benign finding or a definite diagnosis. A more accurate explanation is that the duplication is a susceptibility CNV with reduced penetrance and variable expressivity (Tang et al. 2025). In practice, the CNV result should be interpreted in the context of fetal ultrasound findings. Parental testing, if available, helps determine whether the duplication is inherited from an apparently unaffected parent. Formal classification still needs to follow ACMG/ClinGen standards and should be checked against public databases and available clinical evidence (Tang et al. 2025). An inherited duplication from a healthy parent may be reassuring, but it does not eliminate risk. A de novo duplication or a duplication accompanied by significant fetal structural abnormalities may warrant discussion of a broader range of possible outcomes during counseling. Among the 5 fetuses that underwent pedigree validation in this study, 1 Case was de novo, and the remaining 4 Cases were inherited from phenotypically normal parents. Following comprehensive genetic counseling, the parents of all five fetuses elected to continue their pregnancies. This observation reflects the individual decisions of these families and should not be interpreted as a general recommendation regarding pregnancy management. No obvious abnormalities were reported during postnatal follow‐up.

The prenatal significance of an isolated 16p13.11 microduplication remains difficult to predict because of its reduced penetrance and variable expressivity. Pregnancy management should be individualized and decided by the parents after balanced genetic counseling and, when appropriate, multidisciplinary consultation. Our findings do not provide a basis for a uniform recommendation regarding pregnancy management in an individual pregnancy. Most live‐born children with follow‐up data showed no obvious abnormalities, but the prognosis of an individual fetus remains difficult to predict. Parents should be informed of both the possibility of a favorable outcome and the residual risk of neurodevelopmental or congenital abnormalities. After birth, follow‐up should be guided by the child's clinical course rather than by the CNV result alone. We monitor speech and motor development closely, watch for learning or behavioral concerns as the child grows, and arrange cardiac review when indicated. Some of these concerns may only emerge later in childhood or adulthood, which is why ongoing surveillance matters (Cai et al. 2022). The results should be read in light of several limitations. This was a retrospective single‐center study with a limited number of cases. Parental origin analysis was not systematically available for all cases, which restricted evaluation of inheritance and genotype–phenotype segregation. Follow‐up was mainly based on telephone interviews and parental reports, without standardized neurodevelopmental assessment for every child. Two live‐born children were lost to follow‐up, and follow‐up duration varied across cases. For the terminated pregnancies, systematic fetal autopsy and additional genetic testing were not performed, limiting assessment of the prenatal ultrasound abnormalities.

## 5 Conclusion

In summary, this study provides additional prenatal and postnatal data on fetuses with 16p13.11 microduplication. Most live‐born children with available follow‐up had no apparent abnormalities, while one child showed language and motor developmental delay and one had ventricular septal defect after birth. These findings support cautious interpretation of 16p13.11 microduplication as a susceptibility CNV with reduced penetrance and variable expressivity. In prenatal counseling, this CNV should be evaluated together with fetal imaging, CNV characteristics, parental origin, family history, and plans for postnatal follow‐up, rather than being used as an isolated indication for pregnancy termination.

## Author Contributions

**Xiaoxiao Lv:** conceptualization, methodology, data curation, investigation, formal analysis, visualization, writing – review and editing, writing – original draft, funding acquisition. **Shufei Li:** resources, investigation, software. **Lili Zhou:** formal analysis, validation. **Ruipu Wang:** resources, investigation. **Yuan Yu:** resources, investigation. **Chenyang Xu:** formal analysis, validation. **Xueqin Xu:** formal analysis, visualization. **Xiaohua Tang:** formal analysis, validation. **Huanzheng Li:** conceptualization, methodology, validation, supervision, resources, funding acquisition, project administration, writing – review and editing.

## Funding

This research was supported by the Wenzhou Municipal Bureau of Science and Technology (Self‐raised Funds; Project No. YC20250109) and the Zhejiang Provincial Medical and Health Science and Technology Project (Grant No. 2023KY1155).

## Conflicts of Interest

The authors declare no conflicts of interest.

## Data Availability

The data supporting the findings of this study are available from the corresponding author upon reasonable request.
